# Lung and Infection CT-Scan-Based Segmentation with 3D UNet Architecture and Its Modification

**DOI:** 10.3390/healthcare11020213

**Published:** 2023-01-10

**Authors:** Mohammad Hamid Asnawi, Anindya Apriliyanti Pravitasari, Gumgum Darmawan, Triyani Hendrawati, Intan Nurma Yulita, Jadi Suprijadi, Farid Azhar Lutfi Nugraha

**Affiliations:** 1 Department of Statistics, Faculty of Mathematics and Natural Sciences, Universitas Padjadjaran, Bandung 45363, Indonesia; 2 Department of Computer Science, Faculty of Mathematics and Natural Sciences, Universitas Padjadjaran, Bandung 45363, Indonesia

**Keywords:** COVID-19 CT-scan, 3D image segmentation, 3D UNet, 3D ResUNet, 3D VGGUNet, 3D DenseUNet

## Abstract

COVID-19 is the disease that has spread over the world since December 2019. This disease has a negative impact on individuals, governments, and even the global economy, which has caused the WHO to declare COVID-19 as a PHEIC (Public Health Emergency of International Concern). Until now, there has been no medicine that can completely cure COVID-19. Therefore, to prevent the spread and reduce the negative impact of COVID-19, an accurate and fast test is needed. The use of chest radiography imaging technology, such as CXR and CT-scan, plays a significant role in the diagnosis of COVID-19. In this study, CT-scan segmentation will be carried out using the 3D version of the most recommended segmentation algorithm for bio-medical images, namely 3D UNet, and three other architectures from the 3D UNet modifications, namely 3D ResUNet, 3D VGGUNet, and 3D DenseUNet. These four architectures will be used in two cases of segmentation: binary-class segmentation, where each architecture will segment the lung area from a CT scan; and multi-class segmentation, where each architecture will segment the lung and infection area from a CT scan. Before entering the model, the dataset is preprocessed first by applying a minmax scaler to scale the pixel value to a range of zero to one, and the CLAHE method is also applied to eliminate intensity in homogeneity and noise from the data. Of the four models tested in this study, surprisingly, the original 3D UNet produced the most satisfactory results compared to the other three architectures, although it requires more iterations to obtain the maximum results. For the binary-class segmentation case, 3D UNet produced IoU scores, Dice scores, and accuracy of 94.32%, 97.05%, and 99.37%, respectively. For the case of multi-class segmentation, 3D UNet produced IoU scores, Dice scores, and accuracy of 81.58%, 88.61%, and 98.78%, respectively. The use of 3D segmentation architecture will be very helpful for medical personnel because, apart from helping the process of diagnosing someone with COVID-19, they can also find out the severity of the disease through 3D infection projections.

## 1. Introduction

COVID-19 is an infectious respiratory disease caused by SARS-CoV-2 (Severe Acute Respiratory Syndrome Corona Virus 2). This disease has spread over the world since December 2019; it started in one of the cities in China, namely Wuhan, and caused a global pandemic [1,2]. COVID-19 is recognized as a global pandemic because this disease is a highly contagious disease that has caused the WHO (World Health Organization) to declare this COVID-19 disease a PHEIC (Public Health Emergency of International Concern). This is due to the fact that this disease has a significant negative impact on individuals, governments, and even the global economy [3,4,5,6]. COVID-19 patients experience symptoms ranging from asymptomatic to symptomatic, including illness, lethargy, fever, cough, loss of smell and taste, and even the potentially fatal ARDS (Acute Respiratory Disease Syndrome) [7]. COVID-19 mostly affects the lungs, causing lung infection, but it can also induce intestinal infections, resulting in digestive symptoms such as nausea, vomiting, and diarrhea [8].

From 2019 to now, there has still been no medical treatment that has been proven to cure COVID-19 in its entirety [9]. Therefore, one of the most needed precautions to reduce the spread of this COVID-19 virus is accurate and fast testing. The most common COVID-19 disease detection technique used worldwide, and considered the gold standard for testing for COVID-19, is RT-PCR (Reverse Transcription-Polymerase Chain Reaction) [9,10,11]. In just 4 to 6 h, RT-PCR is able to identify the presence or absence of SARS-CoV-2 RNA from respiratory specimens obtained by nasopharyngeal or oropharyngeal swabs [12]. However, the drawbacks of the RT-PCR test are that this test requires a lot of medical personnel to perform it manually, and each country has a limited stock of RT-PCR test kits. Furthermore, The RT-PCR test has a fairly low sensitivity (range 70 to 90) [13], which causes a high false-negative rate. This is due to many factors, including sample preparation and quality control that is not very mature due to time pressure and the situations around the world that are getting worse [14,15,16]. In addition to diagnosis using the RT-PCR test, there are also other diagnoses that use chest radiography imaging, namely CXR (Chest X-Ray) and CT-scan (Computed Tomography Scans). Both of these have proven to be more accurate than RT-PCR, but it is necessary for a radiologist to identify and look for radiological signs that show COVID-19 symptoms on the image. Although CXR diagnosis is generally less expensive, faster, and exposes the patient to less radiation than CT-scan [17,18], CT-scan is more recommended for more accurate diagnostic results than CXR.

CT-scan is one of examination tools to diagnose the existence of COVID-19 symptoms trough radiological images [8,19]. The CT-scan is preferable because it can overcome the RT-PCR test’s low sensitivity, so that when compared to RT-PCR, CT-scans increase the accuracy and speed of diagnosis [20]. When compared with other chest radiography imaging techniques, specifically CXR, CT-scans are more recommended in the diagnosis of COVID-19 or lung disease in general because CT-scans are not affected by chest tissue, and produce a three-dimensional image output, resulting in better visibility. In addition, one of the significant advantages of CT-scans is their versatility, where CT-scans can diagnose COVID-19 as well as non-covid diseases [16,21,22]. Although CT-scans have proven to be more accurate in diagnosing COVID-19 or lung disease in general, in the diagnostic stage, a radiologist is needed to diagnose and look for radiological signs that show symptoms of COVID-19 on the images. Therefore, it is very necessary to automate the diagnosis of lung disease using CT-scans. In addition to saving time and effort, it is also necessary to avoid errors that occur when performed manually by a radiologist, since the diagnosis depends on the accuracy and experience of the radiologist. Image segmentation is one of the keys in the stage of COVID-19 disease diagnosis automation. With image segmentation, in addition to being useful for knowing the required object segmentation area, it can also be useful for knowing more about the characteristics of a disease from the segmented object area used. Therefore, it is very important to know and find an image segmentation algorithm for medical images, especially CT-Scans, that is effective in helping medical experts diagnose COVID-19 diseases accurately and quickly [23].

With the rapid developments in the field of machine learning, many machine learning algorithms are used for medical image processing needs. One of the deep learning algorithms that is currently the most widely used for medical image processing, or even image processing in general, is the CNN (Convolutional Neural Network) algorithm. CNN has been widely used to diagnose other diseases such as tumors, malaria, cancer, and so on [24,25,26,27]. These studies confirm that CNN can also be used to detect the COVID-19 disease. In this study, the 3D UNet architecture and other three 3D UNet modification architectures will be used to segment the lung (binary-class segmentation), and the lung and infection (multi-class segmentation), on CT-scans. Seven models will be compared for each segmentation case (binary and multi-class). The seven models are: pure 3D UNet, 3D ResUNet (ResNet152) without transfer learning, 3D ResUNet (ResNet152) with transfer learning, 3D VGGUNet (VGG19) without transfer learning, 3D VGGUNet (VGG19) without transfer learning, 3D DenseUNet (DenseNet201) with transfer learning, and 3D DenseUNet without transfer learning. The reason for using the 3D UNet architecture and its modification, apart from the fact that the CT-scan data is already in 3D form, is that 3D UNet was chosen so that the image preprocessing and postprocessing processes are simpler. That is different from when we use UNet (2D), with which it is necessary to first separate each slice in the 3D image into one 2D image. Even though the use of the 3D UNet architecture requires larger and more expensive computational resources than UNet (2D), by using 3D UNet, we take advantage of the volume in the image and still consider the image as a single 3D image.

The following are the research’s main contributions:

1. To effectively analyze CT-scan images, which are three-dimensional (3D), we have employed 3D deep learning architectures that are capable of analyzing data in a single 3D unit. This approach is distinct from previous research on this problem, where most studies have utilized two-dimensional (2D) architectures that require slicing the 3D image into individual 2D slices. The implementation of 3D architectures can improve the efficiency of CT-scan analysis by eliminating the need for preprocessing before model input and streamlining the post-processing stage through the ability to seamlessly project 3D images.
2. In this research, we have modified the 3D UNet architecture by replacing the encoder with three classification architectures: 3D VGG19, 3D ResNet152, and 3D DenseNet201. This resulted in three distinct image segmentation architectures: 3D VGGUNet, 3D ResUNet, and 3D DenseUNet. To determine which architecture is the most effective, we will compare the performance of these architectures using five evaluation metrics: IoU score, Dice score, accuracy, and F1-score.
3. By using a 3D segmentation architecture on a CT-scan, in addition to being able to help the COVID-19 disease diagnosis process, the 3D output generated from the model can help medical personnel determine the severity of the disease, such as mild, moderate, or severe, through a 3D infection projection that can be easily seen from the output model that generates a file in 3D shape.

The remainder of this paper is structured as follows: In Section 2, several research papers related to the diagnosis of COVID-19 using chest radiography imaging in general and the use of semantic segmentation in CT-scan for COVID-19 diagnosis in particular are discussed. The dataset, data preprocessing, proposed architecture in this study, metrics evaluation, and model setting that were used in this study are explained in Section 3. In Section 4, the result of each proposed architecture is explained and discussed. Finally, Section 5 concludes the paper.

## 2. Related Works

This section contains works related to our research, including general works on the diagnosis of COVID-19 using chest radiography imaging and the use of semantic segmentation in a specific CT-scan dataset. These works serve as the foundation for our investigation into efficient and effective methods for analyzing CT-scans for COVID-19 diagnosis using image segmentation techniques.

Because of the rapid advancement of technology, several researchers are contributing to the creation of a COVID-19 diagnosis system that uses artificial intelligence with chest radiography imaging media, including CXR and CT-scan. For the use of CXR media, many researchers diagnose COVID-19 using classification methods. In [28], various deep learning architectures, namely ResNet18, ResNet50, SqueezeNet, and DenseNet121, are used for CXR classification, and most of these networks achieve a specificity rate of around 90% and a sensitivity rate of 98%. In [29], COVID-Net was used to classify CXR images into COVID-19, non-COVID, bacterial infection, and normal. Moreover, in [30], high-level features were extracted using various ImageNet pre-trained models, and then all those features were fed into SVM to classify the COVID-19 cases. In addition to using the classification method on CXR, some researchers also use the image segmentation method. For example, in [31], researchers achieve state-of-the-art performance and can provide clinically interpretable saliency maps, which are very useful for COVID-19 diagnosis. In addition, in [32], researchers also applied the image segmentation method on CXR to extend it to cases with extreme abnormalities. CT-scans, in addition to CXR, have been shown in previous studies to be an extremely useful tool for diagnosing COVID-19 [33,34,35]. Practitioners and doctors use CT abnormalities that correspond to COVID-19. It has been discovered that CT scans show discrete patterns that can be used to identify infected individuals even in the early stages, making automatic CT medical imaging analysis an attractive topic of research among researchers [35]. It has also been discovered that CT diagnosis for COVID-19 anomaly detection can be performed prior to the onset of clinical symptoms [36]. As a result, several research papers have been proposed for the automatic early detection of COVID-19 using the classification and segmentation method infection on CT images [37,38]. Table 1 presents a summary of the method we will use and various previous studies regarding segmentation on CT-scan with the same dataset, namely the COVID-19 CT Lung and Infection Segmentation Dataset [39].

Owais et al. [40] introduced DMDF-Net (Dual Multiscale Dilated Fusion Network); this architecture is tailored for precise and fast segmentation of lung and infection areas from CT-scans, and it achieved an IoU score of 67.22%, a Dice similarity coefficient of 75.7%, an average precision of 69.92%, a specificity of 99.79%, a sensitivity of 72.78%, an enhance-alignment of 91.11%, and an MAE of 0.026. The use of CLAHE preprocessing and only cropping the area of interest was carried out by Mahmoudi et al. [9] to improve the image segmentation results when using the UNet architecture, resulting in a Dice score of 98% and 91% for the lung and infection segmentation tasks, respectively. Wang et al. [41] developed a new segmentation architecture, called SSA-Net, with the aim of automatically identifying areas of infection on CT-scans of the lungs. This architecture’s main idea is to utilize a self-attention mechanism to broaden the receptive field and improve representation learning by extracting useful contextual information from deeper layers without additional training. Wang et al. [41] conducted experiments using SSA-Net on various datasets, and on dataset [39] obtained an average Dice similarity coefficient of 75.4%. Punn et al. [42] introduced the CHS-Net (COVID-19 hierarchical segmentation network); two RAIU-Net are connected in series in this architecture, and this architecture achieved an accuracy of 96.5%, a precision of 75.6%, a specificity of 96.9%, a recall of 88.5%, a Dice coefficient of 81.6%, and Jaccard similarity of 79.1%. Yin et al. [10] modified the UNet architecture by combining the SA module and the Dense SAPP module so as to create a new architecture called SD-UNet. This architecture achieved the metrics of Jaccard similarity, specificity, accuracy, Dice similarity coefficient, and sensitivity of 77.02% (47.88%), 99.32% (99.07%), 99.06% (98.21%), 86.96% (59.36%), and 89.88% (61.69%), respectively, for the binary-class (multi-class) segmentation. In study conducted by Singh et al. [43], UNet was used again to segment CT-scans. In this study Singh et al. replaced the UNet backbone (encoder) to EfficientNetB0, and it achieved a sensitivity of 84.5%, a specificity of 93.9%, and Dice coefficient of 65%. 

Previous research on image segmentation for COVID-19 CT scans has utilized various approaches, many of which utilize convolutional neural networks (CNNs). However, these approaches often rely on 2D architectures, which can lengthen the modeling process, due to the need for data preprocessing to fit the data for 2D architectures and post-processing to project the predicted results into a 3D shape. To address this issue, we propose a solution using 3D CNN architectures in our research. Specifically, we use the 3D version of the well-known UNet architecture for image segmentation, resulting in the 3D UNet architecture. Additionally, we modify the 3D UNet architecture by using three classification architectures, namely VGG 19, ResNet 152, and DenseNet 201, resulting in the 3D VGGUNet, 3D ResUNet, and 3D DenseUNet architectures.

## 3. Materials and Methods 

This section contains our proposed approach and the materials that we will use in this study. We start by describing the dataset that will be used in this study. After that, we will explain what pre-processing stages are applied to the data, the proposed method or architecture that will be used in this study, the metrics evaluation that will be used in the evaluation of our models, and at the end, the model setting for every architecture will be explained, too.

### 3.1. Dataset

In this study, the lung CT-scan dataset of Ma et al. [39] was used for the CT-scan segmentation modelling (training and testing) process. This dataset consists of 20 CT-scans of COVID-19 patients collected from radiopaedia [44] and the corona-cases initiative (RAIOSS) [45]. In addition to providing CT-scan files, ref. [39] also provides three masks for segmentation purposes, namely ‘lung mask’, ‘infection mask’, and ‘lung and infection mask’. In the work of Ma et al. [46], it is explained that this dataset was manually annotated by two radiologists and verified by an experienced radiologist. Table 2 presents an overview of the CT-scan dataset used.

In this study, segmentation will be carried out on the “lung mask” and the “lung and infection mask” in each model. Two segmentation cases were carried out to test the strength of each model in the cases of binary-class segmentation (lung mask) and multi-class segmentation (lung and infection mask).

Each CT-scan from the [39] dataset has a different width and height, a depth (slice), and a different level of infection severity. Table 3 shows the more detailed profile of each patient’s CT scan used.

### 3.2. Data Preprocessing

Since we want to use 3D image segmentation architecture, we need to adjust the width, height, and depth of each image to the same size. In this study, we adjusted each CT-scan data to 128 × 128 × 128. The resizing process for each CT-scan data is assisted by ImageJ software, and in the process of resizing the depth of each data, ImageJ applies average upsampling and average downsampling with bilinear interpolation.

Two preprocessing steps will be carried out on the CT-scan image file: scaling the pixel value and applying the CLAHE method. The image scaling process is carried out on each pixel value in the CT-scan into a value with a range of 0 to 1. This scaling stage is carried out using the minmax scaler method.

Furthermore, the CLAHE (Contrast Limited Adaptive Histogram Equalization) method is applied to overcome the contrast problems (noise and intensity inhomogeneity). CLAHE was used to intensify the contrast of the obtained images [47]. This method is a variant of AHE (Adaptive Histogram Equalization). CLAHE’s main objective is to determine the mapping for each pixel based on its neighborhood grayscale distribution using a transformation function that reduces contrast amplification in densely packed areas. In [48,49], CLAHE has shown its effectiveness in allocating displayed intensity levels in chest CT-scans. In Table 4, a comparison of the CT-scan slices before CLAHE was applied and after CLAHE was applied is shown.

For the case of binary-class segmentation, the lung mask pixels consisting of 0: background, 1: left lung, and 2: right lung are changed to 0: background and 1: lung. The unification of the left lung and right lung masks is not only performed to create a binary-class segmentation case, but it is also performed to facilitate the learning model process because there is no significant difference in the image between the left lung and right lung. The unification of left lung and right lung is also carried out on the lung and infection mask for multi-class segmentation, where, in this multi-class segmentation, the pixel lung and infection mask consist of 0: background, 1: left lung, 2: right lung, and 4: infection is changed to 0: background, 1: warp, and 2: infection. 

### 3.3. Network Architecture

This work proposes four segmentation network architectures, namely 3D UNet [50], 3D ResUNet, 3D VGGUNet, and 3D DenseUNet. Each architecture will be applied in both binary-class CT-scan segmentation (lung segmentation) and multi-class CT-scan segmentation (lung and infection segmentation). For the 3D ResUNet, 3D VGGUNet, and 3D DenseUNet architectures, two experiments will be carried out for each segmentation, using transfer learning and not using transfer learning. From this, a total of seven models will be obtained for each segmentation case.

**Figure 1:**
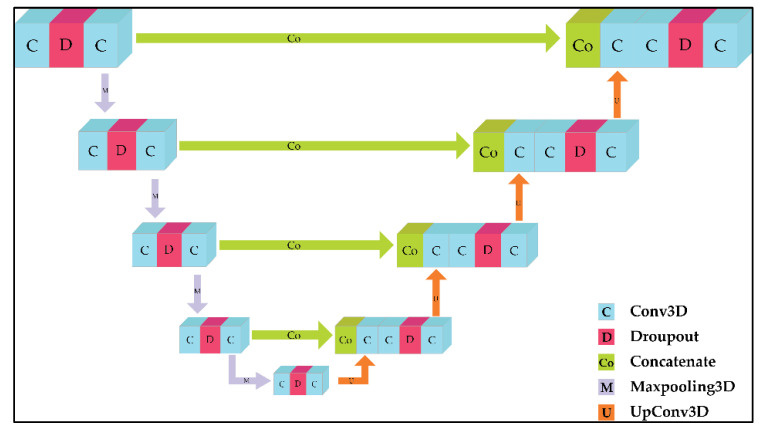
3D UNet architecture.

3D UNet has two main parts, namely the encoder and decoder. The encoder part, also called the contracting part, is in charge of extracting global features from the image. The encoder consists of convolution blocks (consisting of batch normalization, ReLu) and max pooling for downsampling. The decoder part, also known as the expanding path, consists of upconvolution, a concatenation layer with a feature map from the encoder part, and convolutional blocks. To avoid overfitting, a dropout layer is added to each convolutional block. The UNet 3D architecture is shown in Figure 1.

**Figure 2:**
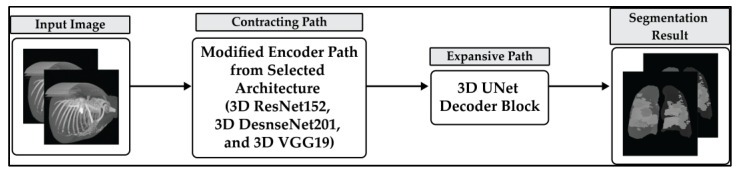
The general segmentation process with scenario of 3D U-Net modified architectures.

The 3D ResUNet, 3D VGGUNet, and 3D DenseUNet architectures are modifications of the 3D UNet architecture. These three architectures replace the encoder portion of 3D UNet with 3D ResNet, 3D VGG, and 3D DenseNet, respectively. 3D ResUNet uses the 3D ResNet152 architecture to replace the encoder part of 3D UNet because the ResNet152 version is the latest version of the 3D ResNet version series available (ResNet18, ResNet34, ResNet50, ResNet152). As with 3D VGGUNet, the latest version of the 3D VGG architecture, namely 3D VGG19, is used as the backbone or encoder of the 3D UNet architecture. The same goes for 3D DenseUNet. DenseNet201’s 3D architecture was chosen for the reason of being the most up-to-date version compared to other 3D DenseNet versions (3D DenseNet121, 3D DenseNet169, and 3D DenseNet201). The schematic of these 3 architectures is not much different from the 3D UNet shown in Figure 1. Figure 2 shows the general segmentation process of the 3D VGGUNet, 3D ResUNet, and 3D DenseUNet architectures.

The 3D versions of the VGG and ResNet classification architectures were chosen to modify the encoder part of the 3D UNet architecture because these two architectures are some of the most widely used in research, with the ResNet architecture being used in over 142,000 studies and the VGG architecture being used in over 119,000. Apart from being widely used, these two architectures were chosen because both of them have proven to be very good at solving classification problems, as evidenced by their wins in the ImageNet 2014 (VGG) and ImageNet 2015 (ResNet) competitions. The 3D version of the DenseNet architecture was chosen in this study because it is one of the architectures that has recently begun to be widely used, because it has many advantages, such as reducing the vanishing-gradient problem, strengthening feature propagation, encouraging feature reuse, and having parameters that are not too large. This architecture is the development of the most widely used classification architecture, namely ResNet. In the previous study by Alalwan et al. [51], 3D DenseUNet was used to segment liver and tumors from CT-scans, but the DenseNet version of the 3D DenseUNet architecture used in Alalwan et al.’s [51] study is a DenseNet version with a depth of 169. This study will use a deeper version of Densenet, namely DenseNet201. Due to the deep structure of each architecture, visualization will not be possible. Further details on the modified architectures used in this study can be found in Appendix A.

In this study, each modified architecture will be trained using both traditional training and transfer learning approaches. Transfer learning is a machine learning technique in which patterns learned from a pre-trained model are utilized to improve the performance of a model on a new task [52]. This can be particularly useful when working with a small training and testing dataset, as it allows us to leverage the knowledge and experience of the pre-trained model. In this research, we will apply transfer learning to the encoder portion of the architecture, including the 3D VGG19, 3D ResNet152, and 3D DenseNet201. The weights for the transfer learning process will be obtained from models that have been trained on the ImageNet dataset, a large and widely-used dataset for training and evaluating deep learning models. By applying transfer learning and utilizing the knowledge of these pre-trained models, we hope to improve the performance of our modified architectures on the CT-scan image segmentation task.

### 3.4. Metrics Evaluation

In this study, we use five evaluation metric indices to evaluate the performance of each network: IoU (Intersection Over Union score, also known as the Jaccard Index), DSc (Dice Score, or also known as the F1-score and Sørensen–Dice coefficient), Acc (Accuracy), Pre (Precision), and Rec (Recall). In the case of image segmentation, IoU and Dsc are the most frequently used metrics and are recommended to evaluate the model [53,54]. In general, the Dsc and IoU are used to see the similarity of the results of the segmentation area between the predicted result and the ground truth. The IoU and Dsc formulas are defined as follows:

(1) $$IoU=\frac{\left|A_{1}\cap A_{2}\right|}{\left|A_{1}\cup A_{2}\right|}$$

(2) $$Dsc=\frac{2\left|A_{1}\cap A_{2}\right|}{\left|A_{1}\right|+\left|A_{2}\right|}$$

From Equations (1) and (2), it should be noted that *A*_1_ denotes the ground truth, and *A*_2_ denotes the predicted result by the model.

In addition to using IoU and Dsc, this study also used two classification metrics: accuracy, which measures the ratio of correctly identified predicted pixels to all predicted pixels, and F1 score, which is calculated as the harmonic mean of precision and recall. Precision measures the accuracy of predictions by calculating the ratio of true positive predicted pixels to the total number of positive predictions, and recall measures completeness by calculating the ratio of true positive predicted pixels to the total number of actual positive pixels. The accuracy and F1 score are defined as follows:

(3) $$Acc=\frac{TP+TN}{TP+FP+TN+FN}$$

(4) $$F1\;score=\frac{2TP}{2TP+FP+FN}$$

Here, *TP (True Positive)* denotes the number of the lung pixel or infected pixel being correctly identified, *TN (True Negative)* denotes the number of uninfected pixels or non-lung pixels being correctly identified, *FP (False Positive)* represents the number of infected pixels or lung pixels being wrongly identified as the uninfected or non-lung pixels, and *FN (False Negative)* represents the number of the non-lung pixels or the uninfected pixels being wrongly identified as lung or infected pixels.

### 3.5. Experimental Setting

**Figure 3:**
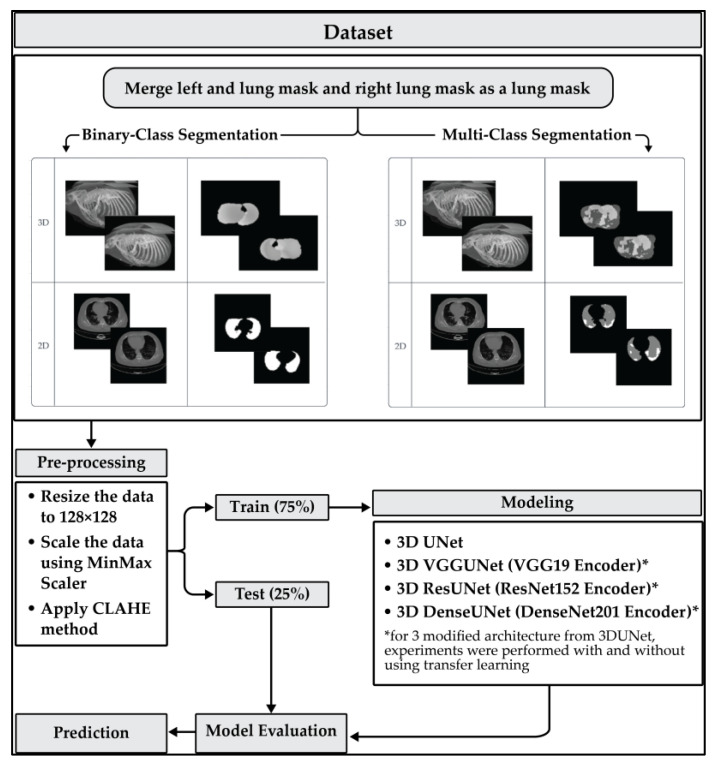
The modeling schemes.

All models are trained using the ADAM optimizer with a learning rate of $1\times 10^{-4}$. To maximize the learning capabilities of each architecture, we set the maximum epoch to 5000 with an early-stop patience of 250. The loss functions used in the training process are total Dice loss and focal loss. Mixing between focal loss and Dice loss is performed because, after several experiments, the segmentation results using total loss from Dice loss and focal loss are better than using only Dice loss or only using focal loss. The total loss here is obtained by adding up the Dice loss with the focal loss, where each class weight for the Dice loss is set equal. Hold-out validation is used for the process of training and testing models. The data is split by 75% for model training and 25% for model testing. We run all models in this study using Google Colab Pro, with a GPU as a hardware accelerator and high-RAM usage for runtime shape. Figure 3 depicts the modeling scheme used in this study.

## 4. Results

In this section, the results of the binary-class and multi-class segmentation experiments on the CT-scan will be shown. As described previously, the case of binary-class segmentation will be applied to segment the lungs from the CT-scan, while the case of multi-class segmentation will be applied to segment the lungs and infection from the CT-scan. We will see the results of the evaluation of metrics from 3D UNet, 3D VGGNet without transfer learning, 3D Res UNet without transfer learning, 3D DenseUNet without transfer learning, 3D VGGUNet with transfer learning, 3D ResUNet with transfer learning, and 3D DenseUNet with transfer learning in each CT-scan segmentation case.

### 4.1. Lung Segmentation (Binary-Class Segmentation)

For the purpose of comparison, the same hyperparameter values have been set, and the same distribution of training and testing data sets is used for the modeling process in each architecture. Table 5 shows the results of the evaluation metrics for each architecture in lung segmentation.

**Figure 4:**
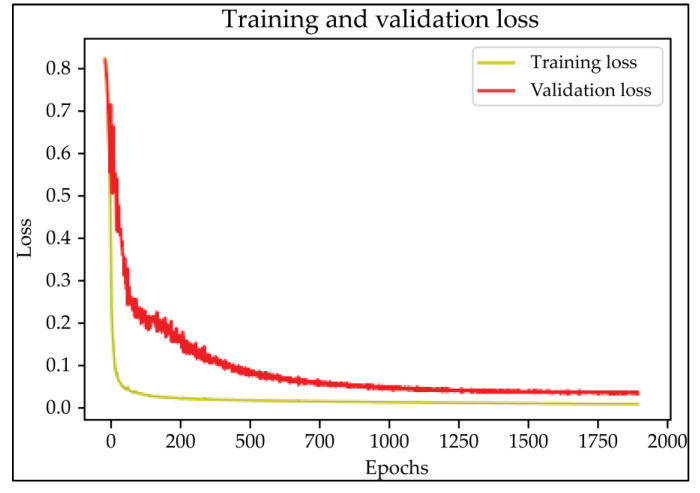
The training and validation loss curve of the 3D UNet architecture CT-scan binary-class segmentation learning process.

Based on the results of the evaluation metrics for each architecture in Table 5, surprisingly, 3D UNet is better than the other six methods. Compared with the 3D VGGUNet architecture with transfer learning, which achieved the second best result on average, 3D UNet improved by 0.37%, 0.23%, 0.03%, and 0,19% in IoU score, Dice score, accuracy, and F1-score, respectively. Although 3D UNet has the best evaluation of metrics compared to other architectures, 3D UNet has the longest maximum learning iteration process of 1663, in contrast to other architectures, which are modifications of 3D UNet, and which have an average maximum learning iteration of 278. Of the seven models that have been tried, 3D DenseUNet obtained first place as the architecture with the fastest learning time, ±4459 s and ±6539 s without transfer learning and using transfer learning, respectively. The 3D UNet architecture stays in the second last position with a learning process time of ±17,217 s, and for the position of the architecture that has the longest training process, it is the 3D VGGUNet, with transfer learning reaching ±23,200 s. The comparison of loss training and testing on the 3D UNet learning process for the lung segmentation is shown in Figure 4. Furthermore, in Table 6, the comparison of ground truth and the prediction results of the 3D UNet model in 2D (slice) and 3D projections for this binary-class segmentation case can be seen.

### 4.2. Lung and Infection Segmentation (Multi-Class Segmentation)

Similar to binary-class segmentation, in the case of multi-class segmentation, the same hyperparameter values and training testing data distribution have been established, with the intention of comparing each architecture. Table 7 shows the results of the evaluation metrics for each architecture in lung and infection segmentation.

**Figure 5:**
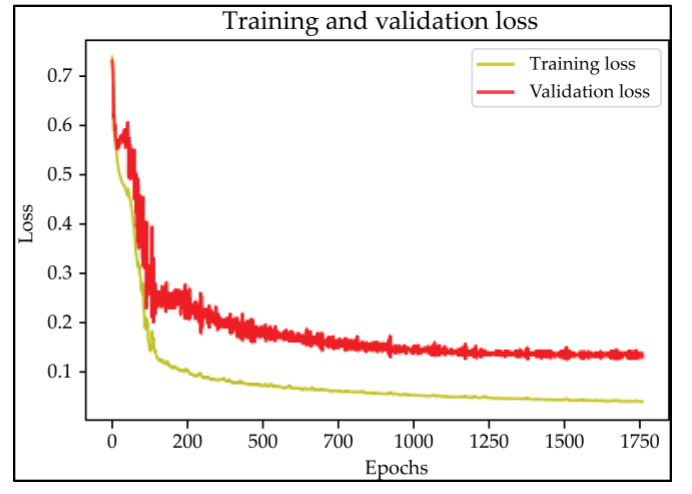
The training and validation loss curve of the 3D UNet architecture CT-scan binary-class segmentation learning process.

Based on the results of the evaluation metrics for each architecture in Table 6, similar to the lung segmentation case, in the lung and infection segmentation case, 3D UNet is better than the other six methods. Compared with the 3D VGGUNet architecture with transfer learning, which achieved the second-best result on average, 3D UNet improved by 8.18%, 7.39%, 0.38% and 0.38% in IoU score, Dice score, accuracy, and F1-score, respectively. Although 3D Unet has the best evaluation of metrics compared to other architectures, 3D UNet has the longest maximum learning iteration process of 1510, in contrast to other architectures, which are modifications of 3D UNet, and which have an average maximum learning iteration of 233. Of the seven models that have been tried, 3D DenseUNet without transfer learning obtained first place as the architecture with the fastest learning time, specifically ±4602 s. The 3D UNet architecture stays in the second to last position with a learning process time of ±12320 s, and in the position of the architecture that has the longest training process is the 3D VGGUNet with transfer learning, reaching ±8702 s. The comparison of loss training and testing on the 3D UNet learning process for the lung and infection segmentation is shown in Figure 5. Furthermore, in Table 8, the comparison of ground truth and the prediction results of the 3D UNet model in 2D (slice) and 3D projections in this multi-class segmentation case can be seen.

### 4.3. Result Discussion

With the aim of experimenting using the 3D version of the UNet architecture and comparing it with its three modifications, namely 3D VGGUNet, 3D ResUNet, and 3D DenseNet, in the case of binary-class (lung) segmentation and multi-class (lung and infection) segmentation. Surprisingly, the original 3D UNet performs much better in both segmenting the binary-class and the multi-class than the modified 3D UNet. Although, on average, the modified architecture of 3D UNet does not perform as well as the original 3D UNet, the three modified architectures have much faster maximum learning iterations than the original 3D UNet, which is on average below 300 epochs, while for the original 3D UNet it requires more than 1500 epochs to reach the maximum learning iteration. In the case of the modified architectures of 3D UNet, it is also seen that using transfer learning on those three architectures increases the model performance compared to without using transfer learning.

In the case of lung segmentation, which can be seen in Figure 2, the 3D UNet architecture studied the case very well, and there was no indication of overfitting or underfitting in the model. In the case of binary-class segmentation, 3D UNet produces IoU scores, Dice scores, accuracy, and F1-score of 94.32%, 97.05%, 99.37%, and 97.07%, respectively. In this lung segmentation case, if sorted based on the results of the metrics evaluation, it was found that 3D ResUNet became the architecture with the lowest average evaluation metrics, followed by 3D DenseUNet, 3D VGGUNet, and the original 3D UNet, with the best average metrics evaluation from three other architectures.

In the case of multi-class segmentation, as shown in Figure 3, the 3D UNet architecture studies lung and infection segmentation cases quite well. Although not as well as when studying lung segmentation cases, the graph in Figure 3 shows that both lines of training loss and validation loss are close enough that we can assume that the model does not indicate under or over fitting. In the case of lung and infection segmentation, 3D UNet scored 81.58%, 88.61%, 98.78%, and 98.78% for the metrics IoU score, Dice score, accuracy, and F1-score, respectively. Similar to binary-class segmentation, in this multi-class segmentation, 3D UNet gets the best average metrics evaluation, followed by 3D VGGUNet, 3D DenseUNet, and 3D ResUNet, as the architectures with the lowest average metrics evaluation of the other three architectures.

In general, these four architectures obtain acceptable evaluation results for predicting the lung or the lung and infection segmentation because, in addition to preprocessing the data, these four 3D architectures take advantage of the volume/depth of the CT-scan as a single data unit when it is entered into the model in the learning process, in contrast to when using a 2D architecture, which only considers one slice as a single data unit when it is entered into the model in the learning process. It should be noted that in this study, the mask/ground truth from the original data was modified by unifying the left and the right lung. Because of that, the cases used in this study differed from cases used in previous studies, despite the fact that they used the same dataset or same general goal, which is to assist in the process of diagnosing COVID-19 by segmenting images that take advantage of technological advances.

## 5. Conclusions

In this study, we applied the 3D version as well as three modifications of one of the most used and most recommended architectures for biomedical images, namely 3D UNet, 3D VGGUNet, 3D ResUNet, and 3D DenseUNet, for cases of COVID-19 CT-scan segmentation. All architectures were applied in two cases: binary-class segmentation to segment the lung from CT-scan, and multi-class segmentation to segment the lung and infection from CT-scan. To try and find the best results in the COVID-19 CT-scan segmentation, transfer learning was applied to each of the three modified architectures. The preprocessing operations were also performed on the dataset, namely resizing the height, length, and depth to the same size, with the aim that the data could entered into the 3D architecture, and applying the CLAHE method to the dataset to clarify the data and make it easier for the network to study each case. The experimental result shows that although the 3D UNet has a very large maximum iteration, the 3D UNet has better performance than the other three modified architectures. In the case of lung segmentation, 3D UNet produces very accurate segmentation predictions with an IoU score of 94.32% and a Dice score of 97.05%, and in the case of lung and infection segmentation, 3D UNet also produces a fairly accurate prediction with an IoU score of 81.58% and a Dice score of 88.61%. In general, the 3D UNet architecture gets good results, not only because of the preprocessing that is performed, but also because this architecture utilizes the volume or depth of 3D data. This study proves that UNet’s 3D architecture can have a major impact on learning, technological developments, and the diagnosis of COVID-19. However, one of the shortcomings in this study is the limited dataset of COVID-19 labeled CT-scans. This causes insufficient training and testing of data for all models. In the future, this study could be expanded in the following aspects: explore various parameter tunings for each architecture; modify and/or add other blocks to the architecture; and, of course, reapply these architectures to more and larger datasets to obtain better COVID-19 diagnosis performance through CT-scan segmentation.

## Figures and Tables

**Table 1:** A summary of the recently published studies on image segmentation using the same dataset.

Method	Summary
DMDF-Net [40]	DMDF-Net (Dual Multiscale Dilated Fusion Network) is proposed to produce robust segmentation of small lesions in CT images. To achieve superior segmentation performance, this architecture utilizes the power of multiscale deep feature fusion within the encoder and decoder modules in a mutually beneficial manner.
UNet [9]	The UNet architecture is used for precise and fast segmentation of lung and infection areas from CT-scan. CLAHE and cropping were also used in the preprocessing to remove the noise and only use the lung area (region of interest) from each slice.
SSA-Net [41]	SSA-Net (Spatial Self-Attention Network) was created with the aim of automatically identifying areas of infection on CT scans of the lungs. SSA-Net utilizes a self-attention mechanism to broaden the receptive field and improve representation learning by extracting useful contextual information from deeper layers without additional training. In addition, this architecture introduces a spatial convolution layer to accelerate training convergence and strengthen the network.
CHS-Net [42]	CHS-Net (COVID-19 hierarchical segmentation network) is proposed to identify the COVID-19 infected area from CT-scan. In this architecture, two models of RAIU-Net (Residual Attention Inception U-Net) are connected in series, where in the first model a contour map of the lung will be generated and the second model will identify the infected area.
SD-UNet [10]	SD-UNet, this architecture is the modified UNet architecture that combines the SA (Squeeze and Attention) with the Dense ASPP (Dense Atrous Spatial Pyramid Pooling) module. In this architecture, the SA module is used to fully exploit the global context information and strengthen the attention of pixel grouping. The Dense ASPP is used to capture the multi-scale of COVID-19 lessons.
UNet-EfficientB0 [43]	Using EfficientNetB0 as the backbone (encoder) on the UNet architecture
Various 3D UNet (Proposed)	We used the 3D UNet architecture in this study, as well as various types of backbone (encoder) on the 3D UNet architecture using no transfer learning and transfer learning. The backbones being tested in this study are 3D ResNet152, 3D VGG19, and 3D DenseNet201.

**Table 2:** Three samples (patient 1, patient 2, and patient 20) from the used dataset.

3D Projection of CT-Scan	CT-Scan Slice	Lung Mask	Infection Mask	Lung and Infection Mask
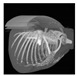	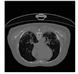	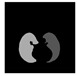	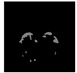	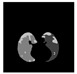
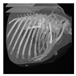	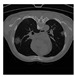	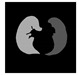	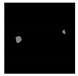	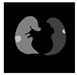
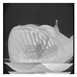	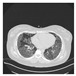	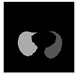	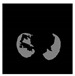	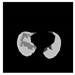

**Table 3:** Source, infection severity, and size information for each patient’s CT-scan.

Patient	Source	Infection Severity	Size (Width × Height × Depth)
Patient 1	RAIOSS	Moderate	512 × 512 × 301
Patient 2	RAIOSS	Mild	512 × 512 × 200
Patient 3	RAIOSS	Severe	512 × 512 × 200
Patient 4	RAIOSS	Mild	512 × 512 × 270
Patient 5	RAIOSS	Mild	512 × 512 × 290
Patient 6	RAIOSS	Moderate	512 × 512 × 213
Patient 7	RAIOSS	Moderate	512 × 512 × 249
Patient 8	RAIOSS	Moderate	512 × 512 × 301
Patient 9	RAIOSS	Moderate	512 × 512 × 256
Patient 10	RAIOSS	Severe	512 × 512 × 301
Patient 11	Radiopaedia	Severe	630 × 630 × 39
Patient 12	Radiopaedia	Severe	630 × 630 × 45
Patient 13	Radiopaedia	Moderate	630 × 630 × 39
Patient 14	Radiopaedia	Moderate	630 × 630 × 418
Patient 15	Radiopaedia	Severe	630 × 401 × 110
Patient 16	Radiopaedia	Moderate	630 × 630 × 66
Patient 17	Radiopaedia	Mild	630 × 630 × 42
Patient 18	Radiopaedia	Mild	630 × 630 × 42
Patient 19	Radiopaedia	Mild	630 × 630 × 45
Patient 20	Radiopaedia	Severe	630 × 630 × 93

**Table 4:** Comparison before and after applying CLAHE preprocessing to CT-scan.

Without CLAHE	CLAHE
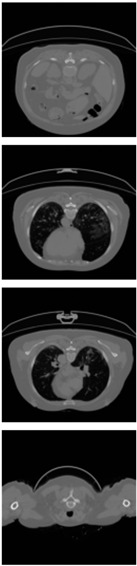	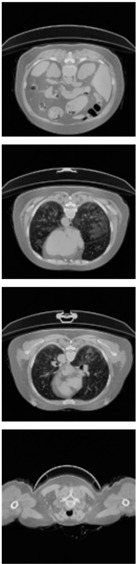

**Table 5:** The IoU Score, Dice Score, accuracy, F1-score epoch of learning, and time per epoch results of each architecture for the CT-scan binary-class segmentation.

	Architecture	IoU	DSc	Acc	F1	Epoch ^1^	Time
	3D UNet	**0.9432**	**0.9705**	**0.9937**	**0.9707**	1663	±9 s/epoch
Without Transfer Learning	3D VGGUNet	0.9287	0.9624	0.9920	0.9630	230	±24 s/epoch
	3D ResUNet	0.8325	0.9072	0.9793	0.9086	357	±14 s/epoch
	3D DenseUNet	0.9204	0.9580	0.9912	0.9585	93	±13 s/epoch
With Transfer Learning	3D VGGUNet	0.9395	0.9682	0.9934	0.9688	330	±40 s/epoch
	3D ResUNet	0.9183	0.9569	0.9909	0.9574	405	±14 s/epoch
	3D DenseUNet	0.9260	0.9610	0.9919	0.9616	253	±13 s/epoch

^1^ The number of epochs is obtained after reducing the total training epochs with a patience value of early stopping. The bolded numbers in the table indicate the highest values compared to other architectures.

**Table 6:** Comparison between ground truth and prediction results of lung segmentation with 3D UNet architecture.

	Original CT-Scan	Ground Truth	Prediction
3D Projection	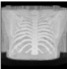	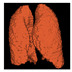	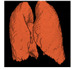
Slice (2D)	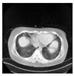	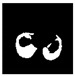	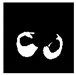
	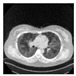	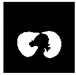	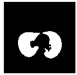
	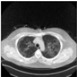	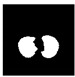	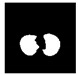

**Table 7:** The IoU Score, Dice Score, accuracy, and F1-score, epoch of learning, and time per epoch results of each architecture for the CT-scan multi-class segmentation.

	Architecture	IoU	DSc	Acc	F1	Epoch ^1^	Time
	3D UNet	**0.8158**	**0.8861**	**0.9878**	0.9878	1510	±7 s/epoch
Without Transfer Learning	3D VGGUNet	0.7276	0.8049	0.9839	0.9839	146	±24 s/epoch
	3D ResUNet	0.7089	0.7839	0.9833	0.9833	151	±14 s/epoch
	3D DenseUNet	0.7143	0.7916	0.9825	0.9826	104	±13 s/epoch
With Transfer Learning	3D VGGUNet	0.7340	0.8122	0.9840	0.9840	600	±24 s/epoch
	3D ResUNet	0.7381	0.8178	0.9832	0.9832	208	±19 s/epoch
	3D DenseUNet	0.7193	0.7960	0.9835	0.9836	189	±13 s/epoch

^1^ The number of epochs is obtained after reducing the total training epochs with a patience value of early stopping. The bolded numbers in the table indicate the highest values compared to other architectures.

**Table 8:** Comparison between ground truth and prediction results of lung and infection segmentation with 3D UNet architecture.

	Original CT-Scan	Ground Truth	Prediction
3D Projection	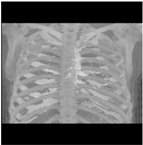	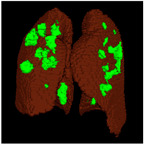	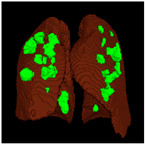
Slice	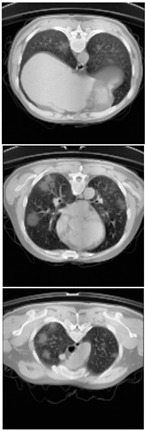	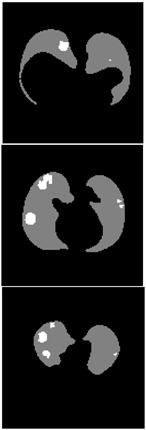	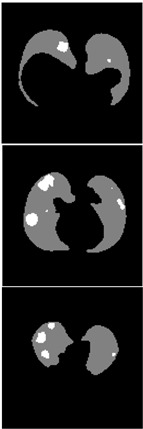

## Data Availability

Data is available at https://doi.org/10.5281/zenodo.3757476 [39], accessed on 18 November 2022.

## Appendix A

### Modified Architectures Details

Appendix A gives details structures for each modified architectures used in this study.

**Table A1:** Details on 3D VGGUNet Architecture.

	Block Type	Block Info	Details on Each Bock/Sub-Block
Encoder part (DenseNet201)	Convolutional Block	1st and 2nd sub-block	Layers: Convolutional 3D + Convolutional 3D + Max Pooling 3D
		3rd–5th sub-block	Layers: Convolutional 3D + Convolutional 3D + Convolutional 3D + Convolutional 3D + Max Pooling 3D
	Convolutional Block	Contains two sub-block	Layers: Convolutional 3D + Batch Normalization + ReLu
Decoder part	Convolutional Block	Contains two sub-blocks	Layers: Up Sampling 3D, Batch Normalization, ReLu, Concatenate, Convultional 3D, Batch Normalization, ReLu
	Output	-	Layers: Convolutional 3D + Softmax/Sigmoid

**Table A2:** Details on 3D ResUNet Architecture.

	Block Type	Block Info	Details on Each Bock/Sub-Block
Encoder part (DenseNet201)	Input Block	-	Layers: Batch Normalization + Zero Padding 3D + Batch Normalization + ReLu + Zero Padding 3D + Max Pooling 3D
	Convolutional Block	1st sub-block	Layers: Convolutional 3D + Batch Normalization + ReLu + Zero Padding 3D + Convolutional 3D + Batch Normalization + ReLu + Convolutional 3D + Convolutional 3D + Concatenate
		2nd and 3rd sub-block	Layers: Convolutional 3D + Batch Normalization + ReLu + Zero Padding 3D + Convolutional 3D + Batch Normalization + ReLu + Convolutional 3D + Concatenate
	Convolutional Block	1st sub-block	Layers: Convolutional 3D + Batch Normalization + ReLu + Zero Padding 3D + Convolutional 3D + Batch Normalization + ReLu + Convolutional 3D + Convolutional 3D + Concatenate
		2nd–8th sub-block	Layers: Convolutional 3D + Batch Normalization + ReLu + Zero Padding 3D + Convolutional 3D + Batch Normalization + ReLu + Convolutional 3D + Concatenate
	Convolutional Block	1st sub-block	Layers: Convolutional 3D + Batch Normalization + ReLu + Zero Padding 3D + Convolutional 3D + Batch Normalization + ReLu + Convolutional 3D + Convolutional 3D + Concatenate
		2nd–36th sub-block	Layers: Convolutional 3D + Batch Normalization + ReLu + Zero Padding 3D + Convolutional 3D + Batch Normalization + ReLu + Convolutional 3D + Concatenate
	Convolutional Block	1st sub-block	Layers: Convolutional 3D + Batch Normalization + ReLu + Zero Padding 3D + Convolutional 3D + Batch Normalization + ReLu + Convolutional 3D + Convolutional 3D + Concatenate
		2nd and 3rd sub-block	Layers: Convolutional 3D + Batch Normalization + ReLu + Zero Padding 3D + Convolutional 3D + Batch Normalization + ReLu + Convolutional 3D + Concatenate
Decoder part	Convolutional Block	Consists of five sub-blocks	Layers: Batch Normalization + Up Sampling 3D + Concatenate + Convolutional 3D + Batch Normalization + ReLu + Convolutional 3D
	Output	-	Layers: Convolutional 3D + Softmax/Sigmoid

**Table A3:** Details on 3D DenseUNet Architecture.

	Block Type	Block Info	Details on Each Bock/Sub-Block
Encoder part (DenseNet201)	Input Block	-	Layers: Batch Normalization + Convolutional 3D + Batch Normalization + Convolutional 3D + ReLu + Concatenate
	Convolutional Block	Consists of six sub-blocks	Layers: Batch Normalization + ReLu + Convolutional 3D + Batch Normalization + Relu + Convolutional 3D + Concatenate
	Pooling Block	-	Layers: Batch Normalization + ReLu + Convolutional 3D + Average Pooling 3D
	Convolutional Block	Consists of 12 blocks	Layers: Batch Normalization + ReLu + Convolutional 3D + Batch Normalization + Relu + Convolutional 3D + Concatenate
	Pooling Block	-	Layers: Batch Normalization + ReLu + Convolutional 3D + Average Pooling 3D
	Convolutional Block	Consists of 48 blocks	Layers: Batch Normalization + ReLu + Convolutional 3D + Batch Normalization + Relu + Convolutional 3D + Concatenate
	Pooling Block	-	Layers: Batch Normalization + ReLu + Convolutional 3D + Average Pooling 3D
	Convolutional Block	Consists of 32 blocks	Layers: Batch Normalization + ReLu + Convolutional 3D + Batch Normalization + Relu + Convolutional 3D + Concatenate
Decoder part	Convolutional Block	Consists of five blocks	Layers: Up Sampling 3D + Concatenate + Convolutional 3D + Batch Normalization + ReLu
	Output	-	Layers: Convolutional 3D + Softmax/Sigmoid
